# A Radar-Based Contactless System for Joint Phonocardiogram Reconstruction and Cardiac State Segmentation Using a Self-Attention 1D U-Net

**DOI:** 10.3390/s26103151

**Published:** 2026-05-15

**Authors:** Giulio Montanari, Marco Mura, Pasquale Di Viesti, Elia Vignoli, Giorgio Guerzoni, Giorgio Matteo Vitetta

**Affiliations:** 1 Department of Engineering “Enzo Ferrari”, University of Modena and Reggio Emilia, 41125 Modena, Italy; giulio.montanari@unimore.it (G.M.); 260688@studenti.unimore.it (M.M.); giorgio.guerzoni@unimore.it (G.G.); giorgiomatteo.vitetta@unimore.it (G.M.V.); 2 Deep Radars S.r.l., 41125 Modena, Italy; elia.vignoli@deepradars.com

**Keywords:** radar sensing, contactless monitoring, time-series analysis, phonocardiogram reconstruction, cardiac state segmentation, biomedical signal processing, deep learning, self-attention mechanism, 1D U-Net

## Abstract

Contactless vital signs monitoring is becoming increasingly relevant in scenarios where conventional sensors are impractical or not recommended. In this manuscript, a radar-based contactless system for the joint reconstruction of phonocardiogram (PCG) waveforms and cardiac state segmentation is illustrated. The proposed method exploits a self-attention one-dimensional (1D) U-Net fed by a pre-processed radar-derived input to estimate a PCG-like waveform, its envelope, and the four main cardiac phases: S1, systole, S2, and diastole. The accuracy of our method has been assessed on a public synchronized radar–PCG dataset acquired by means of a 24 GHz Doppler radar and a digital stethoscope. On the test subset, the proposed model achieved a 13.4885 dB reduction in log-spectral distance relative to the radar input signal, indicating a marked improvement in waveform fidelity. Segmentation performance also improved, with Micro-$F_{1}$ increasing from 74.41% to 84.17% and Macro-$F_{1}$ from 68.40% to 80.43% on average. Experimental results demonstrated the viability of real-time low-power embedded hardware deployment for contactless auscultation and continuous cardiac monitoring applications. The findings confirm that respiratory interference and low-amplitude signals complicate S2 detection, especially when exacerbated by subject motion.

## 1. Introduction

Continuous vital signs monitoring (VSM) is crucial for assessing the status of any patient and enabling early diagnosis. Conventional contact-based sensors, such as electrocardiogram (ECG), photoplethysmogram (PPG), or seismocardiogram (SCG), may be unsuitable for patients with severe burns or acute mental illnesses or infants [1], and are often unfeasible or prohibited during procedures like computerized axial tomography (CAT) or magnetic resonance imaging (MRI) scans [2].

To overcome these limitations, substantial research efforts have been devoted to the development of contactless VSM methods based on cameras [3], lasers [4], air-coupled ultrasound systems [5], and radars [1]. Despite recent progress establishing air-coupled ultrasound as a promising non-contact modality for VSM, its practical implementation is hindered by difficulties in attaining adequate bandwidth, directivity, and acoustic output [5].

Instead, among the various contactless sensors, millimeter wave radars have been shown to provide high sensitivity, while preserving patient privacy [1]. The majority of the research work on such techniques focuses on the estimation of instantaneous or average breath rate (BR) and heart rate (HR), whereas only a few manuscripts concern the development of methods for extracting cardiac acoustic signals, i.e., heart sounds (HS) (such signals are commonly acquired through stethoscopes, and their plots are known as phonocardiograms (PCGs)), from radar measurements [6,7]; note that the interest in the development of a contactless stethoscope (CS) is motivated by its potential immunity to environmental noise and by the fact that cardiac acoustic signals can be interpreted by clinicians through auscultation [8].

More specifically, in [6], a millimeter-wave radar system is employed to measure cardiac mechanical activity and reconstruct an ECG-like waveform using a deep neural network (NN) that learns the mapping from pre-processed radar data to a reference ECG signal. In [9], generative adversarial networks (GANs) are used to synthesize realistic coronary artery disease heart sound segments, thus augmenting existing datasets to improve classification performance. In [10], a U-Net-style NN is introduced to enhance and denoise radar signals, whereas in [7,11], HS are detected and segmented using hidden semi-Markov models (HSMM). Finally, in [12], a radar-to-SCG conversion deep-learning-based approach is proposed for contactless monitoring and detection of cardiovascular conditions.

Unlike in previous work, in this manuscript, the use of self-attention 1D U-Net is proposed for heart sound reconstruction and cardiac phase estimation. Compared with existing solutions, the proposed architecture enables the joint estimation of the PCG waveform from radar measurements and the segmentation of the radar-based reconstructed HS into their four main cardiac phases, supporting contactless auscultation and VSM. Crucially, the model improves segmentation performance with respect to radar-only baselines, bringing the predicted phases into closer agreement with the reference PCG signal. Moreover, its effectiveness is assessed on the basis of established performance metrics.

The remainder of this manuscript is organized as follows: In Section 2, HS and their common breathing artifacts are described; moreover, the required pre-processing steps for both radar and PCG signals are illustrated. In Section 3, the architecture of the adopted NN is analyzed, whereas Section 4 focuses on evaluating the architectural choices and performance of the trained model in different measurement scenarios, highlighting its key improvements in terms of some standard metrics. Finally, Section 5 summarizes the main findings and concludes the paper.

## 2. Heart Sounds Model and Pre-Processing

### 2.1. Heart Sounds Characteristic

Heart sounds originate from the vibrations generated by the closure of cardiac valves and by the surrounding heart structures [13]. Under normal conditions, auscultation with a stethoscope reveals two primary HS, denoted as S1 and S2. Although auscultation typically covers the frequency range [40,520] Hz, electronic recordings show that a substantial portion of the vibrational energy pours out of this band, with a peak of around 20 Hz.

The cardiac cycle consists of a relaxation phase, known as diastole, followed by a contraction phase, referred to as systole, and can be further divided into four main phases: ventricular filling, isovolumic contraction, ventricular ejection, and isovolumic relaxation. The first HS, S1, is generated during the isovolumic contraction phase due to the closure of the atrioventricular valves at the onset of systole. The second HS, S2, originates during the isovolumic relaxation phase as a result of the closure of the aortic and pulmonary valves, marking the end of systole and the onset of diastole. Accordingly, S1 and S2 occur approximately at the beginning and end of systole, respectively. Based on this, the cardiac cycle can be divided into four segments (e.g., see [11]): the S1 sound, the systolic interval between S1 and S2, the S2 sound, and the diastolic interval between S2 and the subsequent S1.

**Figure 1:**
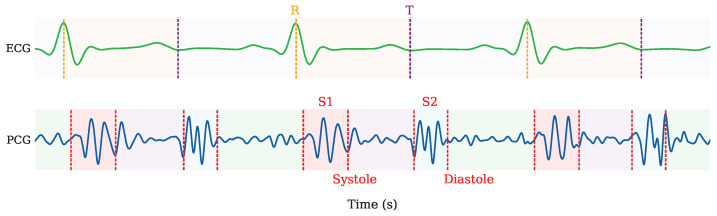
Comparison between PCG measurement with cardiac phases: S1, systole, S2, and diastole and ECG lead signal with R peaks and T wave ends.

Compared with ECG, the gold standard for heart rate (HR) measurement, which records the electrical activity of the heart, the PCG measures the acoustic signals generated during the cardiac cycle. This difference in the measured physical quantity leads to a small, PCG position-dependent time delay between the cardiac vibrations. This effect, combined with the slower propagation speed of sound compared with electrical signals, results in a slight misalignment between ECG and PCG measurements [13]. An example is shown in Figure 1, where the main phases of the ECG and PCG are depicted.

### 2.2. Respiratory Influence

Heart sounds exhibit very low amplitude, as they are associated with small chest vibrations [14], and can be partially masked by breathing harmonics. In addition, respiration can modulate both the amplitude and temporal structure of the HS [7]. The two most common artifacts observed in PCG recordings are described in the following.

#### 2.2.1. Peak Envelope Variation

The amplitudes of the first (S1) and the second (S2) HS exhibit a respiration-dependent modulation referred to as peak envelope variation. Specifically, inspiration and expiration modify the intensity of the valve closures and, consequently, the amplitude of both S1 and S2. This envelope variation is noticeably reduced during breath holding, minimizing amplitude fluctuations [15].

#### 2.2.2. S2 Split

A second well-known respiratory effect is the splitting of S2 into its aortic and pulmonary components. Under normal circumstances, these two components occur nearly simultaneously. During inspiration, however, due to an earlier occurrence of the aortic component and a delay in the pulmonary component, a measurable temporal separation between them, known as S2 splitting, can be observed [15].

For the reasons illustrated above, the extraction of the fundamental components of HS from radar measurements requires the use of devices capable of high range resolution [10] and the application of advanced signal-processing techniques.

### 2.3. Signal Model

Radar-based contactless monitoring exploits the periodic chest displacements induced by cardiorespiratory activity. The instantaneous chest displacement ΔR(t) can be modeled as the superposition of several contributions and, in particular, as [16]

(1) $$\Delta R\left(t\right)=\delta_{r}\left(t\right)+\delta_{h}\left(t\right)+\delta_{\mathrm{hs}}\left(t\right)+\delta_{\mathrm{rbm}}\left(t\right),$$

where $\delta_{r}\left(t\right)$, $\delta_{h}\left(t\right)$, and $\delta_{\mathrm{hs}}\left(t\right)$ represent the displacements associated with respiration, heartbeats, and HS, respectively, while $\delta_{\mathrm{rbm}}\left(t\right)$ is the involuntary random body movement (RBM) acting as noise for VSM.

For a single-input single-output (SISO) continuous wave (CW) Doppler radar, the samples of the complex baseband sequence {x[n]} obtained from in-phase and quadrature (I/Q) demodulation after analog-to-digital conversion can be expressed as [1]

(2) $$\begin{array}{cc} x[n] & =x_{I}\left[n\right]+jx_{Q}\left[n\right]+w\left[n\right]\\ \; & =a\;e^{j\psi [n]}+w\left[n\right],\;n=0,1,\ldots ,N-1, \end{array}$$

where *N* represents the overall number of samples acquired over the considered observation interval, *a* denotes the signal amplitude, w[n] is additive noise, and

(3) $$\begin{array}{cc} \psi [n] & =\frac{4\pi R_{0}}{\lambda }+\frac{4\pi \;\Delta R[n]}{\lambda },\\ \; & =\psi_{0}+\frac{4\pi \;\Delta R[n]}{\lambda }, \end{array}$$

is a phase term depending linearly on chest displacement; here, $\psi_{0}$ is a constant phase offset determined by the nominal radar-to-chest distance, λ is the radar wavelength, $\Delta R\left[n\right]≜\Delta R\left(nT_{s}\right)$ is the chest displacement, and $T_{s}$ is the sampling period.

If we take into consideration the phase variation

(4) $$\Delta \psi \left[n\right]=\frac{4\pi \;\Delta R[n]}{\lambda }$$

only, the associated displacement variation over time can be written as

(5) $$\Delta R\left[n\right]=\frac{\lambda }{4\pi }\Delta \psi \left[n\right].$$

Equation (5) shows that the samples of the sequence {ΔR[n]} associated with chest displacement are linearly related to the samples of the phase variation sequence {Δψ[n]}. Therefore, according to (1), the temporal evolution of {Δψ[n]} contains relevant information about respiratory and cardiac activity, including HS.

### 2.4. Radar and PCG Pre-Processing

**Figure 2:**
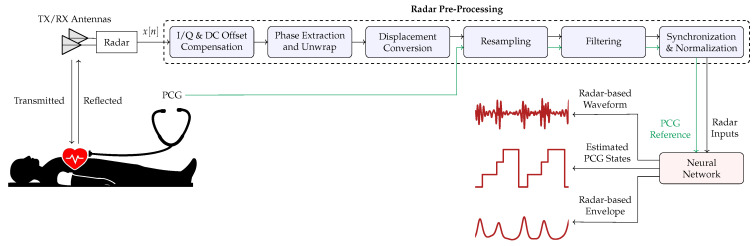
Block diagram describing the signal processing chain adopted in our work. In green the PCG reference signal processing used as ground truth during the NN training.

Prior to their use in training, and subsequent inference, radar and PCG signals need to undergo a pre-processing stage; its main steps, illustrated in Figure 2, are described below.

#### 2.4.1. I/Q and DC Offset Compensation

**Figure 3:**
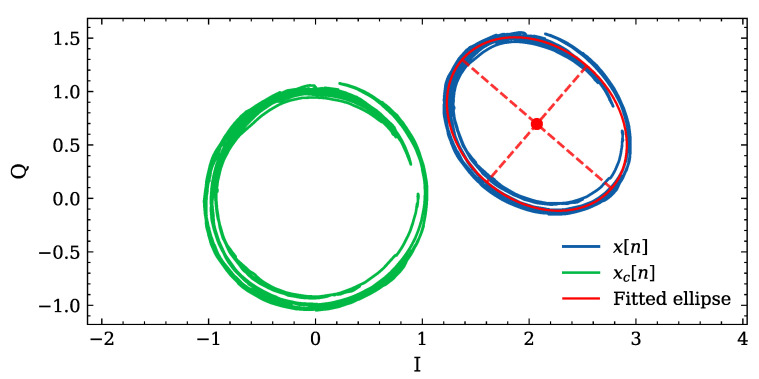
Example of I/Q compensation based on geometric ellipse fitting.

In a real system, the I/Q components of the radar signal are subject to non-idealities; for this reason, the model (see (2))

(6) $$\begin{array}{cc} x_{I}\left[n\right] & =A_{I}\cos\left(\psi [n\right])+O_{I},\\ x_{Q}\left[n\right] & =A_{Q}\sin\left(\psi \left[n\right]+\psi_{e}\right)+O_{Q}, \end{array}$$

is commonly adopted to account for their presence (e.g., see [17]) here, $A_{I}$ and $A_{Q}$ denote the amplitude mismatches, $\psi_{e}$ the phase imbalance, and $O_{I}$ and $O_{Q}$ the in-phase and quadrature DC offsets. Due to system impairments, the I/Q trajectory is described by an ellipse instead of an ideal circle. In the technical literature, a geometric ellipse-fitting method, which estimates the ellipse parameters (center, axes, and orientation) by minimizing the orthogonal distance between the measured samples and the ellipse itself, has been proposed [18]. These parameters are then used to apply an affine transformation for restoring a circular trajectory, as shown in Figure 3; this effectively compensates for amplitude imbalance, phase errors, and DC offsets.

#### 2.4.2. Phase Extraction, Unwrapping, and Displacement Conversion

The compensated I/Q components $x_{c}\left[n\right]$ of the signal provided by a Doppler radar are processed to extract the phase signal Δψ[n] according to (4). Due to the 2π ambiguity of the phase shift Δψ, unambiguous displacement ΔR[n] can only be detected within the range of λ/2. For larger displacements, phase continuity is guaranteed by unwrapping; for this aim, the differentiate and cross-multiply (DACM) algorithm is employed [19]. In place of the direct phase estimate,

(7) $$\psi \left[n\right]=\mathrm{atan}2(x_{c,Q}\left[n\right],x_{c,I}\left[n\right]),$$

wrapped in the interval (−π,π] (and resulting in discontinuities of 2π), the DACM method computes the phase difference

(8) $$\begin{array}{cc} \Delta \psi_{u}\left[n\right] & =\arg\left(x_{c}\left[n\right]x_{c}^{*}\left[n-1\right]\right)\\ \; & =\arg(x_{c,I}\left[n\right]x_{c,I}\left[n-1\right]+x_{c,Q}\left[n\right]x_{c,Q}\left[n-1\right]\\ \; & \;+j(x_{c,Q}\left[n\right]x_{c,I}\left[n-1\right]-x_{c,I}\left[n\right]x_{c,Q}\left[n-1\right])), \end{array}$$

between consecutive samples; this quantity can be also expressed as

(9) $$\begin{array}{cc} \Delta \psi_{u}\left[n\right] & =\mathrm{atan}2(x_{c,Q}\left[n\right]x_{c,I}\left[n-1\right]-x_{c,I}\left[n\right]x_{c,Q}\left[n-1\right],\\ \; & \;x_{c,I}\left[n\right]x_{c,I}\left[n-1\right]+x_{c,Q}\left[n\right]x_{c,Q}\left[n-1\right]). \end{array}$$

**Figure 4:**
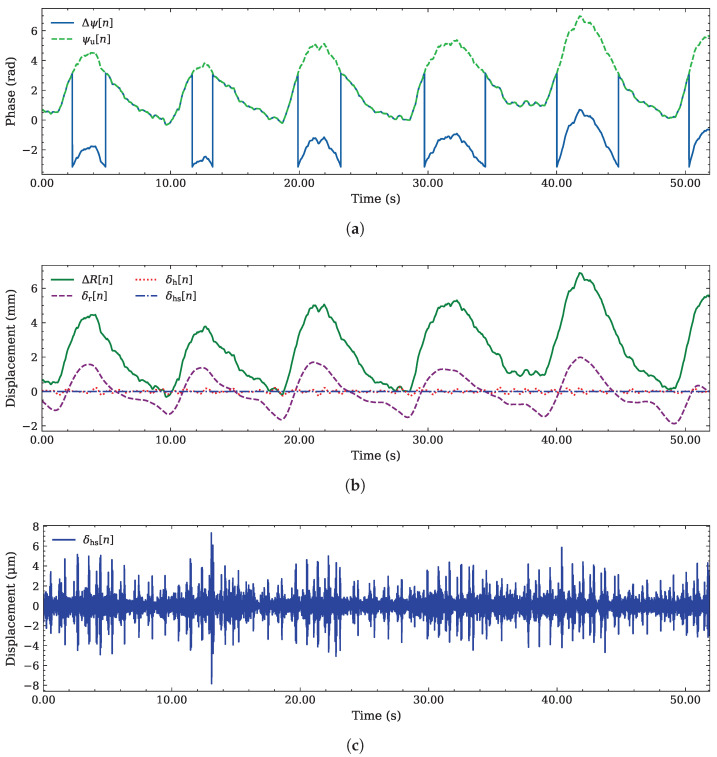
Example of phase extraction, unwrapping, and displacement conversion for the radar signal: (**a**) phase variation Δψ[n] and associated unwrapped phase $\psi_{u}\left[n\right]$ generated by the DACM algorithm; (**b**) total displacement ΔR[n] and its filtered components $\delta_{r}\left[n\right]$, $\delta_{h}\left[n\right]$, and $\delta_{\mathrm{hs}}\left[n\right]$ (associated with respiration, mechanical heart activity, and HS, respectively); (**c**) displacement $\delta_{\mathrm{hs}}\left[n\right]$ due to HS (measured in μm).

Given the sequence of phase differences, the unwrapped phase $\psi_{u}\left[n\right]$ is evaluated as

(10) $$\psi_{u}\left[n\right]=\psi_{u}\left[0\right]+\sum_{k=1}^{n}\Delta \psi_{u}\left[k\right],$$

where $\psi_{u}\left[0\right]$ is the initial phase. The differences between the phase signal extracted through the atan2 operation and that generated by the DACM algorithm are shown in Figure 4a. Then, the use of (5) allows us to recover the chest displacement as

(11) $$\Delta R\left[n\right]=\frac{\lambda }{4\pi }\psi_{u}\left[n\right],$$

i.e., as a linear transformation of the unwrapped continuous phase $\psi_{u}\left[n\right]$. The unfiltered sequence {ΔR[n]} is represented in Figure 4b, together with each of its components appearing on the right-hand side (RHS) of (1). As can be easily inferred from Figure 4b,c, the range variation caused by HS is on the order of μm, whereas the cardiorespiratory components can be measured in terms of mm. Precise handling of these subtle displacements is essential to maintain the integrity of the HS signal.

#### 2.4.3. Resampling, Filtering, Synchronization, and Normalization

**Figure 5:**
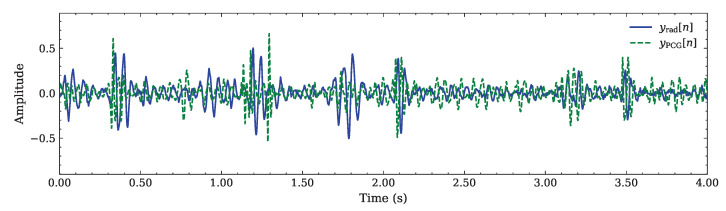
Comparison between the pre-processed radar $\left\{y_{\mathrm{rad}}\left[n\right]\right\}$ and PCG $\left\{y_{\mathrm{PCG}}\left[n\right]\right\}$ sequences.

Both PCG and radar signals are resampled in order to share the same sampling frequency $f_{s}=500$ Hz; then, they are applied to a 5th-order Butterworth band-pass filter attenuating the frequency components outside the [15, 150] Hz range; this removes unwanted spectral components originating from breathing and mechanical heart activity, leaving only the HS. Finally, the resulting signals are synchronized (by matching their timestamps) and normalized to the amplitude range [−1, 1]. This produces the sequences $\left\{y_{\mathrm{PCG}}\left[n\right]\right\}$ and $\left\{y_{\mathrm{rad}}\left[n\right]\right\}$, representing the pre-processed PCG and radar signals, respectively; their time evolution is exemplified in Figure 5.

## 3. Neural Network Architecture

The NN proposed in our work is a multitask self-attention 1D U-Net [20] for PCG audio reconstruction and cardiac phase segmentation. The adopted model is fed by three radar-derived temporal signals and jointly solves three related tasks: PCG waveform reconstruction, envelope estimation, and cardiac phase segmentation. It follows a U-Net-style encoder–decoder architecture, where the encoder learns compact multiscale features from the input signals and the decoder restores temporal resolution for output generation. A self-attention bottleneck is inserted between the encoder and the decoder to capture longer-range dependencies across the cardiac cycle that may be missed by purely convolutional processing. The network uses a shared representation across all tasks so that waveform, envelope, and phase classification information can reinforce each other during training. This design is intended to combine local transient detection, global temporal context, and multitask consistency in a single model.

In particular, the three candidate input synchronized signals are the following:

1. The radar-based HS signal $\left\{y_{\mathrm{rad}}\left[n\right]\right\}$ employed as the primary PCG waveform reconstruction input.
2. Its derivative $\left\{\Delta y_{\mathrm{rad}}\left[n\right]\right\}$ (evaluated as a first-order finite difference). Note that the computation of this sequence aims at emphasizing quick transients such as those related to S1 and S2, improving the segmentation and temporal stability of the reconstructed PCG signal.
3. The unfiltered displacement, ΔR[n]. This represents a slower varying temporal cue employed to preserve low-frequency motion components, smooth out respiratory influences (as described in Section 2.2), and reduce the impact of RBM interference on the network processing.

**Figure 6:**
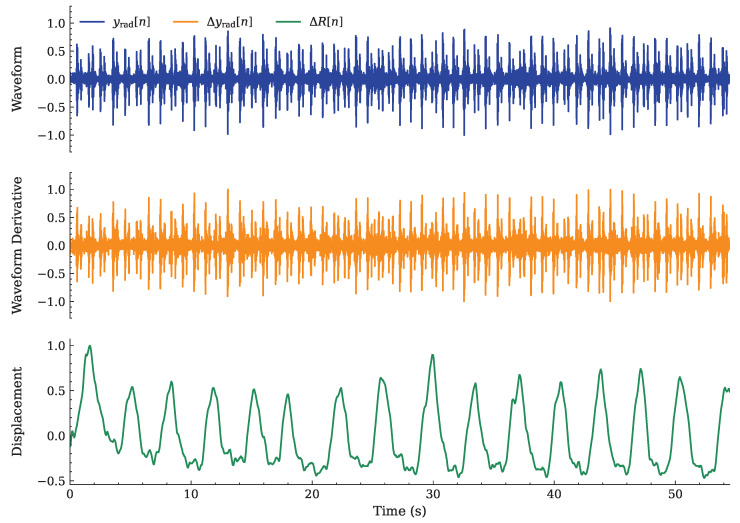
Example of the NN input signals: $\left\{y_{\mathrm{rad}}\left[n\right]\right\}$, $\left\{\Delta y_{\mathrm{rad}}\left[n\right]\right\}$, and {ΔR[n]}.

Each input feature is a block of $n_{s}=2000$ samples acquired over 4 s, at the common sampling frequency $f_{s}=500$ Hz. An example of the input signal before segmentation is shown in Figure 6.

Based on the radar input signals, the following three outputs are provided by the NN:

1. The reconstructed PCG waveform;
2. The four-class HS segmentation, as explained in Section 2.1;
3. The reconstructed radar-derived PCG homomorphic envelope, employed as an auxiliary output to help increase cardiac phase segmentation accuracy [21].

At the NN output, the first and second signals are available in the form of 1×2000 tensors, whereas the four-class HS segmentation signal is shaped as a 4×2000 tensor.

### 3.1. Neural Network Model

The proposed model is a one-dimensional multitask U-Net architecture designed for joint waveform reconstruction, envelope estimation, and cardiac phase segmentation. The network processes a multichannel temporal input and produces three task-specific outputs using a shared latent representation.

The architecture follows a four-level encoder–decoder structure with 24 base channels at the first layer. Given an input window of 4 s sampled at $f_{s}=500$ Hz, the input tensor has dimensions 3×2000. The encoder progressively extracts hierarchical features while reducing temporal resolution, whereas the decoder reconstructs the signal at the original resolution, using skip connections to improve fine-detail reconstruction.

#### 3.1.1. Encoder

Each encoder stage consists of a residual convolutional block, followed by temporal downsampling. The residual block is composed of two one-dimensional convolutional layers with a kernel size of 5 and a padding of 2. The first convolution is followed by group normalization and Gaussian Error Linear Unit (GELU) activation. A dropout rate of 0.2 is applied after the first activation layer to reduce overfitting and improve model generalization during training. A residual shortcut connection adds the input of the block to its output. A final GELU activation is then applied.

Temporal downsampling is performed at the output of the residual block by a one-dimensional convolutional layer with a kernel size of 4, a stride of 2, and unitary padding. Each encoder layer halves the temporal resolution by a factor of 2; for this reason, the size is reduced from 2000 to 125 samples, at the output of the fourth and last encoder. Concurrently, the total number of channels doubles at the output of each encoder stage; this results in an increase from 24 initial channels to 192 at the output of the last encoder.

#### 3.1.2. Transformer Bottleneck

In the bottleneck, the latent representation consists of 125 temporal samples and 192 channels. This representation is projected through a pointwise convolution (1×1 convolution), followed by group normalization and a GELU activation function. This representation is then processed by the transformer bottleneck with an embedding dimension of 192, 8 self-attention heads, 1 transformer encoder layer, and a feedforward network of dimension of 768, followed at the end by a GELU activation. Similar to the encoder, a dropout rate of 0.2 is employed. After the transformer processing, layer normalization is applied, followed by a second 1×1 convolution, ending with group normalization and GELU activation.

The purpose of the self-attention layer is to improve the ability of the model to understand global relationships across the whole time series. While convolutional layers are effective at extracting close temporal features, the self-attention mechanism of the transformer enables direct coherent interaction between distant time steps, through positional encoding. Employing the transformer, specifically at the bottleneck, where the temporal resolution is the lowest, and the extracted feature density is the highest, improves efficiency [22]. This is especially important in the radar-to-PCG waveform reconstruction and segmentation task, in which cardiac-cycle events exhibit long temporally structured patterns, as explained in Section 2.1.

#### 3.1.3. Decoder

The structure of the decoder mirrors the encoder; however, it employs four one-dimensional upsample blocks. Each stage first upsamples the input representation to the corresponding encoder skip connection temporal resolution, with linear interpolation; then, the 1×1 convolution projects the upsampled features onto the output stage number of channels. In contrast with the encoder, each stage doubles the temporal resolution and halves the number of feature channels. Afterwards, the decoder output tensor is concatenated channel-wise with the encoder skip connection, and further refined by a residual convolutional block, employing the same structure as the encoder. The shared representation made available by the last decoder stage is processed by an additional residual refinement block, operating at the original 24 channel dimension, at the full temporal resolution of 2000 samples.

#### 3.1.4. Outputs

**Figure 7:**
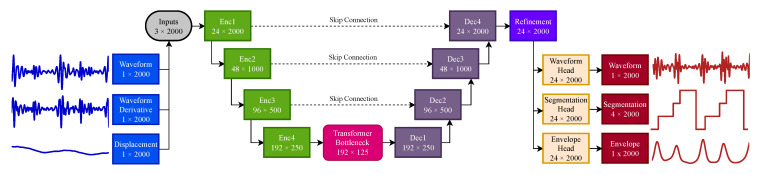
Diagram of the self-attention 1D multitask U-Net architecture.

Both waveform and envelope reconstruction output heads consist of one-dimensional convolutions with a kernel size of 3, mapping 24 channels to 24 channels; this is followed by a GELU activation function and a final 1×1 convolution. This operation maps the 24 channels to a single output channel. The hyperbolic tangent (tanh) activation restricts the output waveform to the normalized [−1, 1] amplitude range. The segmentation head, instead, begins with a 3-tap convolution from 24 to 24 channels; this is followed by GELU and a convolution mapping the 24 channels to 4 output channels, corresponding to the four segmented cardiac phases. A diagram of the neural network architecture is visible in Figure 7, while a brief description of the network architecture is provided in Table 1.

### 3.2. Loss Function

A key aspect of the training of the proposed NN is the choice of the loss function; being a multitask network, this function consists of a weighted sum of each output objective, namely,

(12) $$L=\alpha L_{\mathrm{wav}}+\beta L_{\mathrm{seg}}+\gamma L_{\mathrm{env}},$$

where $L_{\mathrm{wav}}$, $L_{\mathrm{seg}}$, and $L_{\mathrm{env}}$ are the waveform, segmentation, and envelope losses, respectively, with α, β, and γ their respective weights.

The waveform loss

(13) $$L_{\mathrm{wav}}=0.5L_{L}1+0.5L_{\mathrm{STFT}},$$

combines time-domain ($L_{L}1$) and spectral ($L_{\mathrm{STFT}}$) penalties to provide an accurate reconstruction of the radar-based PCG time-series. In practice, the temporal component $L_{L}1$ is computed as the mean absolute error (MAE) between the NN output ${\hat{y}}_{\mathrm{PCG}}\left[n\right]$ and the reference PCG signal $y_{\mathrm{PCG}}\left[n\right]$. To ensure precise signal reconstruction, a multi-resolution magnitude loss $L_{\mathrm{STFT}}$ is employed. This loss is computed for three distinct fast Fourier transform (FFT) sizes, namely, 32, 128 and 512, and corresponding hop sizes 8, 32 and 128, respectively, in order to partly mitigate the STFT trade-off between temporal and spectral resolution, by capturing both short-term and long-term structures.

The segmentation objective

(14) $$L_{\mathrm{seg}}=0.7L_{\mathrm{CE}}+0.3L_{\mathrm{Dice}},$$

is a weighted combination of the normalized cross-entropy (CE) and Dice loss [23]. The former loss is computed in a per-sample fashion and enforces point-wise correct segmentation, improving detection stability. The latter one is a segment-wise measure of the classification overlap between inference and reference, leading to overall increased accuracy.

The imbalance between each of four cardiac phases in terms of duration is handled though per-class weighting. First, the number of samples belonging to each class is accumulated over the desired time window. Then, a per-class (S1, Systole, S2, Diastole) weight vector

(15) $$w_{c}\propto \sqrt{\frac{\bar{n}}{n_{c}}},$$

where $n_{c}$ is the number of samples per class and $\bar{n}$ is the mean count across classes, is computed using the square root of the inverse relative frequency (this results in large values for infrequent classes). The resulting weights are then normalized to obtain the unit mean so that rare classes receive larger penalties in the cost function, while frequent ones are down-weighted without causing instability. These weights are applied to both CE and Dice losses terms of the segmentation loss function $L_{\mathrm{seg}}$ to achieve class balancing.

The last component of the global loss function L (12), i.e., the envelope loss $L_{\mathrm{env}}$, is evaluated as the MAE between the NN reconstructed envelope and the PCG reference envelope.

## 4. Experimental Results

### 4.1. Dataset and Training

The model described in Section 3 and the proposed signal processing chain were trained and evaluated using the dataset presented in [24]. In particular, the dataset consists of synchronized measurements acquired using a radar system operating at 24 GHz [17], a digital stethoscope, an ECG, and a respiration sensor. Data were collected from 11 subjects in various controlled scenarios and at multiple measurement points, including the carotid artery and the back and several frontal positions on the thorax. In total, 265 acquisitions were obtained for a total of approximately 223 min of recordings under different conditions, such as breath hold and post-exercise states and while speaking.

In Table 2, both the mean and standard deviation of the cardiac phase states are presented in terms of time duration and percentage of occupied time. Despite the observed similarities between radar and PCG signals, the baseline radar signal segmentation underestimates S1 and systole measured phases duration, while overestimating S2 and diastole states length on average. These statistics are especially useful during the training step of the model to establish class balance when computing the segmentation loss, as described in Section 3.2.

For training, validation, and testing purposes, the 265 acquisitions dataset, composed of 11 subjects, was split among 9 training subjects and single ones for validation and testing. This protocol avoids leakage between different subject measurements during each step of the process, promoting model generalization. Training was conducted in PyTorch 2.11.0 and Python 3.13 using the AdamW optimizer [25], with a learning rate of $5\cdot 10^{-5}$ and a weight decay of $3\cdot 10^{-5}$, over 50 epochs. A batch size equal to 32 was employed, where each sequence was made of 2000 samples (corresponding to a 4 s acquisition at the sampling frequency $f_{s}=500$ Hz).

### 4.2. Performance Metrics

#### 4.2.1. Radar-to-PCG Waveform Reconstruction

To quantify both the temporal and spectral accuracy of the reconstructed PCG signal, the log-spectral distance (LSD), expressed in dB,

(16) $$\mathrm{LSD}\left(y,\hat{y}\right)=\frac{1}{L}\sum_{l=0}^{L-1}\sqrt{\frac{1}{M}\sum_{m=0}^{M-1}{\left[20\log_{10}\left(\frac{|Y_{l,m}|}{|{\hat{Y}}_{l,m}|}\right)\right]}^{2}}$$

has been employed; here, $Y_{l,m}$ and ${\hat{Y}}_{l,m}$ are the *m*th elements of the STFTs of the reference signal {y[n]} and the estimated signal $\left\{\hat{y}\left[n\right]\right\}$, respectively, and *L* and *M* denote the overall number of time and frequency bins, respectively.

To quantify the similarity between the reference envelope e[n] and the estimated envelope $\hat{e}\left[n\right]$, the Pearson correlation coefficient was computed as

(17) $$r_{\mathrm{env}}=\frac{\sum_{n=1}^{N}\left(e\left[n\right]-\bar{e}\right)\left(\hat{e}\left[n\right]-\tilde{e}\right)}{\sqrt{\sum_{n=1}^{N}{\left(e\left[n\right]-\bar{e}\right)}^{2}}\sqrt{\sum_{n=1}^{N}{\left(\hat{e}\left[n\right]-\tilde{e}\right)}^{2}}},$$

where $\bar{e}$ and $\tilde{e}$ represent their corresponding sample means over the considered signal window.

The envelope correlation metric was included to provide a complementary assessment of reconstruction quality. While LSD quantifies spectral agreement, envelope correlation measures how well the reconstructed signal preserves the temporal amplitude modulation of the reference PCG. In particular, the Pearson correlation coefficient quantifies how closely the estimated envelope follows the reference envelope in terms of their linear relationship. This is particularly relevant for heart-sound signals, since the envelope reflects the relative prominence and timing of the main cardiac acoustic events, such as S1 and S2, and therefore provides an additional indicator of physiological consistency.

#### 4.2.2. Cardiac Phase Segmentation

The segmentation performance of the proposed method has been assessed on the basis of the $F_{1}$ score [11], defined as

(18) $$F_{1}≜2\frac{\mathrm{Precision}\cdot \mathrm{Recall}}{\mathrm{Precision}+\mathrm{Recall}},$$

where

(19) $$\mathrm{Precision}≜\frac{\mathrm{TP}}{\mathrm{TP}+\mathrm{FP}}$$

and

(20) $$\mathrm{Recall}≜\frac{\mathrm{TP}}{\mathrm{TP}+\mathrm{FN}}$$

are evaluated on the basis of sample-wise true positives (TP), false positives (FP), and false negatives (FN). The $F_{1}$ score quantifies the overlap between the model-predicted segmentation and the ground truth annotations provided by the dataset [24]. This metric is bounded between 0 and 1, with a value of 1 indicating perfect prediction of the cardiac phases. In our work, the unweighted average of the $F_{1}$ scores

(21) $${\mathrm{Macro}-F}_{1}≜\frac{1}{4}\sum_{i=1}^{4}F_{1,i}$$

has been also computed to assess the classification performance across the four cardiac phase classes described in Section 2.1. A known limitation of the Macro-$F_{1}$ score is represented by its unweighted nature, which can produce biased results; in particular, large segmentation errors in an underrepresented class may generate misleading results. These considerations have motivated the evaluation of the additional global metric

(22) $${\mathrm{Micro}-F}_{1}≜\frac{2{\mathrm{TP}}_{\mathrm{tot}}}{2{\mathrm{TP}}_{\mathrm{tot}}+{\mathrm{FP}}_{\mathrm{tot}}+2{\mathrm{FN}}_{\mathrm{tot}}}$$

that aggregates true positives, false positives, and false negatives across all classes. This reduces the impact of class imbalance, providing a more representative measure of overall segmentation performance.

### 4.3. Sensitivity Analysis

In order to identify the optimal values of the weights α, β, and γ for the multitask loss functions defined for waveform, segmentation, and envelope, respectively (see (12)), a sensitivity analysis has been carried out. In particular, several combinations were evaluated to investigate the trade-off between waveform reconstruction, cardiac state segmentation, and auxiliary envelope loss. For each combination of weights α/β/γ, the model was trained and tested on the basis of a group split protocol; for each of the 3 tested subjects (5th, 8th and 10th in the dataset), 5 different combinations of training and validation splits were evaluated, resulting in 15 runs for each combination of weights α/β/γ. The computed values of the averaged metrics, defined in Section 4.2, are listed in Table 3.

The results show that all the tested configurations, when compared with the PCG reference signal, substantially improve waveform and segmentation metrics over the radar input baseline. As expected, high values of α result in improved LSD; however, increasing β beyond 0.20 does not yield better segmentation, aside from a small increase in S2 $F_{1}$ score, while also degrading LSD. The weight also fails to improve NN outputs beyond small values.

While no single combination of weights is optimal for all metrics, the 0.70/0.20/0.10 case achieves the highest Macro-$F_{1}$ and is almost tied for the best Micro-$F_{1}$. It also attains low LSD values; this results in a balanced trade-off between waveform fidelity and segmentation performance.

### 4.4. Ablation Analysis

To further test the architecture and the contribution of its features, an ablation analysis has been performed. Our numerical results, in terms of the metrics described in Section 4.2, are listed in Table 4. Note that, in generating these results, the same method as that described in Section 4.3 has been used; for this reason, the values of the mean and standard deviation have been evaluated on the basis of the averaged 3 test subject splits, using 5 different training/validation splits for each. Our results show the following:

- The baseline configuration, employing all three radar-based inputs and the multitask network outputs, achieves a good trade-off between waveform reconstruction and state classification.
- A comparison of the baseline configuration with the other variants of the network highlights that the auxiliary radar HS derivative and the displacement do not significantly contribute to improving metrics. This becomes apparent when the baseline and the radar-HS-input-only variant are juxtaposed; in fact, the first solution outperforms the second one in terms of every measured metric.
- The use of three outputs leads to small, but measurable, benefits in almost all the categories. When the envelope output is disabled, only the S2 $F_{1}$ metric slightly increases; this indicates that envelope supervision is beneficial but not strictly necessary to obtain high overall performance.
- Removing the transformer at the U-Net bottleneck entails a degradation in all aspects across waveform fidelity metrics and segmentation $F_{1}$ scores. This suggests the importance of temporal-context modeling for accurate NN model results.
- The results about single-task variants provide additional insights into the interaction between reconstruction and segmentation objectives. In particular, the segmentation-only model achieves the best segmentation metrics in all states except for S2; this shows that dedicating full model capacity to classification can slightly improve accuracy. However, this comes at the price of losing the reconstruction branch entirely. Conversely, the waveform-only-output variant does not manage to improve on the multitask one and achieves poorer quality. These findings confirm that the proposed multitask architecture offers the most balanced solution for simultaneous radar-to-PCG reconstruction and HS segmentation.

**Table 4:** Ablation-analysis metrics results. Values are reported as mean ± standard deviation; the best mean value for each metric is highlighted in bold.

Variant	LSD [dB]	$r_{\mathrm{env}}$	Micro-$F_{1}$ [%]	Macro-$F_{1}$ [%]	S1 $F_{1}$ [%]	Systole $F_{1}$ [%]	S2 $F_{1}$ [%]	Diastole $F_{1}$ [%]
Radar input	21.7630±1.4906	0.5098±0.0898	77.04±4.91	70.08±6.20	60.86±8.89	75.04±5.59	56.36±7.95	88.07±2.37
Baseline	9.3732±0.2066	0.5792±0.0945	87.82±2.90	83.85±3.65	84.92±3.11	87.68±3.28	69.92±6.68	92.86±1.87
Radar HS only	9.3457±0.3203	0.5810±0.0939	88.29±2.81	84.44±3.68	85.90±2.73	88.08±3.22	70.55±7.38	93.22±1.59
Radar HS only and no envelope	9.3475±0.3455	0.5806±0.0956	88.16±2.92	84.31±3.72	85.24±3.02	87.78±3.38	70.99±7.03	93.24±1.67
Radar HS and displacement	9.2768±0.3092	0.5780±0.0942	88.13±3.12	84.19±4.00	85.35±3.44	87.92±3.48	70.34±7.52	93.17±1.81
No transformer	9.3358±0.3427	0.5745±0.0942	84.95±3.40	80.91±4.11	81.48±3.55	83.90±4.19	67.69±6.73	90.56±2.16
No envelope	9.2691±0.3344	0.5780±0.0963	88.12±2.82	84.23±3.57	85.29±2.90	87.85±3.20	70.59±6.82	93.20±1.64
Waveform only	9.3598±0.5042	0.5706±0.0783	–	–	–	–	–	–
Segmentation only	–	–	88.36±2.92	84.47±3.76	85.64±3.16	88.10±3.23	70.79±7.09	93.34±1.70

### 4.5. Results

Taking advantage of the knowledge gained from the sensitivity and ablation analysis illustrated in Section 4.3 and Section 4.4, respectively, a complete evaluation based on the 11 test-subject dataset has been carried out. The training procedures of the chosen radar-HS-input-only model with 0.70/0.20/0.10 weights follow the protocol presented in Section 4.1, employing a subject-wise split to avoid leakage and improve the generalization of the model. For each of the 11 subjects, 5 different combinations of training and validation were tested, for a total of 55 different runs. A summary of the optimized training hyperparameters is shown in Table 5, while the results obtained for each of the 11 subjects are reported in Table 6.

Overall, the proposed model provides a substantial improvement with respect to the radar HS input baseline. The aggregate LSD decreases, on average, from 23.1625 dB to 9.6740 dB, when compared with the reference PCG waveform, resulting in a 13.4885 dB improvement. Envelope correlation exhibits a mean increase of 0.0980 over the starting radar value, indicating an improvement in the accuracy of the temporal amplitude modulation. In terms of cardiac phase segmentation, Micro-$F_{1}$ improves from 74.41% to 84.17%, while Macro-$F_{1}$ sees an even greater increase, from 68.40% to 80.43%. The $F_{1}$ score improvement is consistent across all cardiac states. The largest relative gains are observed for S1 and S2, starting from the low values of 63.57% and 54.43% at the input, and reaching 80.71% and 68.32%, respectively, at the output of the model. This result is particularly relevant, as the first and second heart sounds correspond to short transient events; this makes their direct identification more difficult. The highest $F_{1}$ average score is achieved by the diastole, with a 89.69% mean score. The most challenging remains S2, due to its lower relative amplitude and artifacts originating from respiratory influence (see Section 2.2).

**Figure 8:**
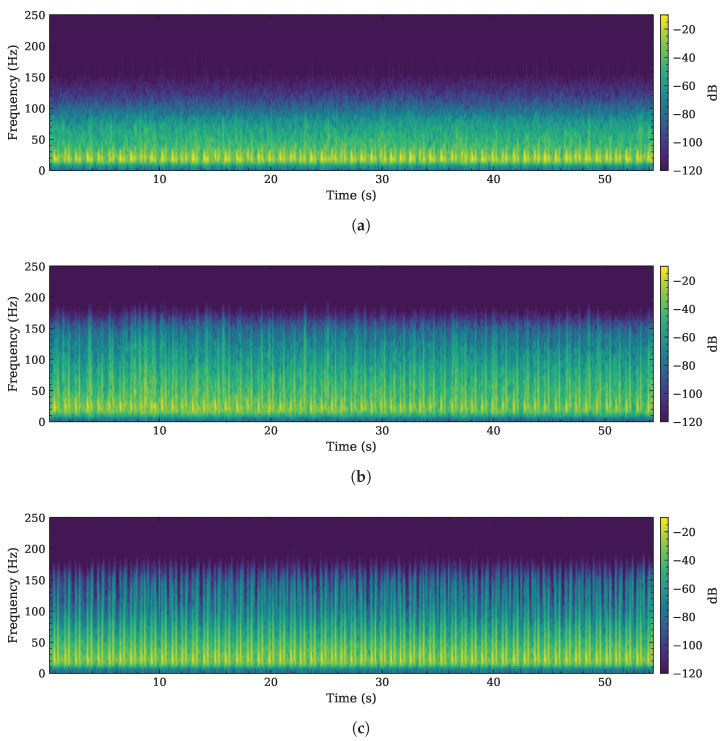
Comparison between the spectrograms of: (**a**) an NN radar-based input signal $y_{\mathrm{rad}}\left[n\right]$; (**b**) the associated PCG reference signal $y_{\mathrm{PCG}}\left[n\right]$; and (**c**) the corresponding PCG signal reconstructed by the proposed self-attention 1D U-Net ${\hat{y}}_{\mathrm{PCG}}\left[n\right]$.

An example of a complete acquisition originating from the dataset provided by the authors of [24] is illustrated in Figure 8. In the original PCG reference (Figure 8b) and in the radar-reconstructed output (Figure 8c), the frequency peaks associated with the S1 and S2 heart sounds (HS) are clearly identifiable. Conversely, these spectral components are less visible in the original radar input signal shown in Figure 8a.

As can be observed in Table 6, subject-wise performance exhibits some variability. Subject 2 achieves the best segmentation metrics, obtaining Micro-$F_{1}$ and Macro-$F_{1}$ equal to 92.13% and 89.41%, respectively, alongside a mean envelope correlation of 0.7099 and an LSD of 8.8436 dB, with respect to the PCG waveform. Worse performance is observed in Subjects 4 and 5, in terms of both waveform reconstruction and cardiac state segmentation.

The final model has been also evaluated by grouping the test recordings according to acquisition scenarios defined in the dataset [24]. Our results are listed in Table 7. The best overall performance is obtained in apnea condition following sport activities, with an LSD of 8.7906 dB, with respect to the PCG reference recording, together with an envelope correlation of 0.6917, and a Micro-$F_{1}$ and Macro-$F_{1}$ of 88.85% and 85.56%, respectively. Good segmentation performance is also observed in the after sport and lying conditions.

The most challenging scenario observed in our experiments is that in which speech is generated during the acquisition of radar measurements; in this case, the highest LSD is found. This suggests that speech-related motion and acoustic disturbances strongly affect the quality of waveform reconstruction. A performance degradation is also observed, even if to a lesser degree, in the distance variation scenario; this highlights the sensitivity of the contactless radar-based HS sensing to the acquisition setup geometry. Motion artifacts induced by the standing posture measurements also reduce segmentation performance when compared with the resting and after sport conditions.

The per-class precision, recall, and $F_{1}$ scores listed in Table 8 show that (a) diastole achieves the best metrics with 90.43% precision, 89.00% recall, and 89.69% $F_{1}$ score; (b) systole and S1 show stable performance; and (c) S2 is the most problematic class, being characterized by the lowest metrics. Note that the challenges with accurate S2 segmentation are consistent with the associated breathing artifacts presented in Section 2.2.

**Figure 9:**
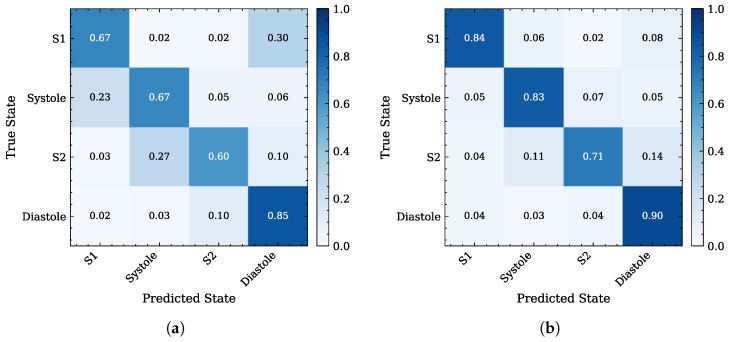
Comparison between the row-normalized confusion matrices of cardiac phase segmentation: (**a**) baseline radar HS input states; (**b**) the output states estimated by the proposed self-attention 1D U-Net. Both signals are compared against the reference PCG segmentation of the dataset.

The row-normalized confusion matrices in Figure 9 further support these observations. Compared with the radar HS input confusion matrix in Figure 9a, the output of the self-attention U-Net in Figure 9b shows a stronger diagonal, indicating improved agreement between predicted and reference PCG cardiac states. The remaining off-diagonal errors mainly involve confusion between neighboring states, especially around the shorter S2 interval, consistent with the lower $F_{1}$ score reported in Table 8.

**Figure 10:**
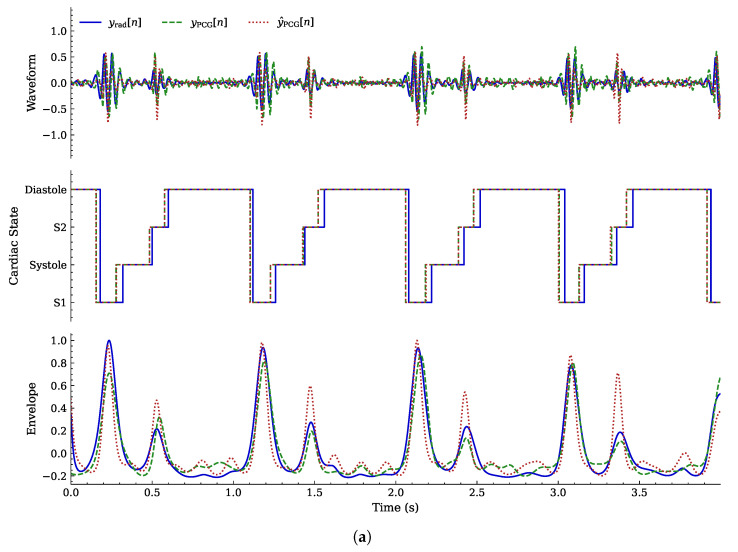

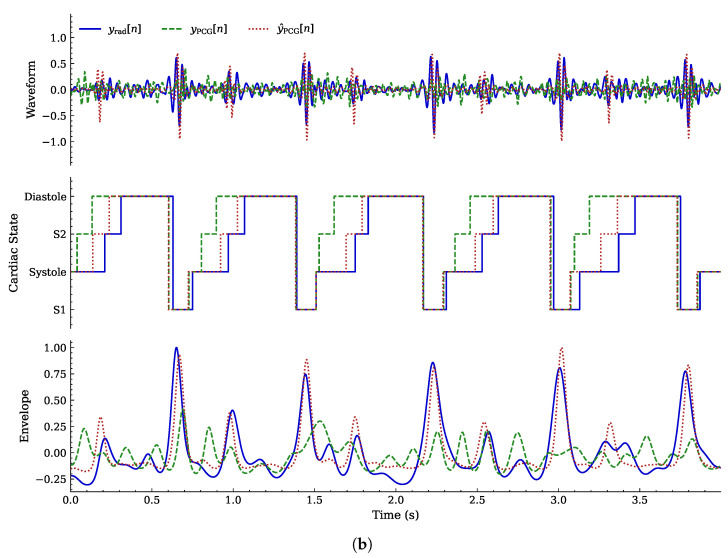
Comparison between two NN outputs generated in response to a 4 s input segment; $\left\{y_{\mathrm{rad}}\left[n\right]\right\}$, $\left\{y_{\mathrm{PCG}}\left[n\right]\right\}$, and $\left\{{\hat{y}}_{\mathrm{PCG}}\left[n\right]\right\}$, referring to waveform, cardiac state segmentation, and envelope reconstruction, respectively, are compared. (**a**) Example of good waveform reconstruction and segmentation during apnea; (**b**) example of unsuccessful PCG and state estimation during speech.

Figure 10 compares successful and unsuccessful waveform reconstruction and cardiac phase segmentation. In the apnea scenario (Figure 10a), the model demonstrates high waveform fidelity and state estimation accuracy. Conversely, during speech (Figure 10b), the model fails to correctly differentiate between states. This failure mode is consistent with the confusion matrix shown in Figure 9b.

### 4.6. Event-Based Comparison

To provide a closer comparison with event-based PCG segmentation studies, an additional S1 and S2 event-detection analysis has been performed using the synchronized ECG reference available in the dataset [24]. Following the evaluation strategy adopted in [11], S1 detections were compared with ECG R-peaks and S2 detections with ECG end-T-wave positions using a ±100 ms tolerance window, corresponding to ±50 samples at $f_{s}=500$ Hz. This event-based metric is distinct from the sample-wise four-state Micro-$F_{1}$ and Macro-$F_{1}$ scores reported in Section 4.5.

The event-based results in Table 9 show that the proposed model improves the combined S1/S2 event-detection $F_{1}$ score with respect to the radar input baseline, mainly through improved S2 localization. The S1 score is comparable to the radar baseline, whereas S2 increases from 79.35% to 88.61%.

A direct comparison with the method presented by Springer et al. in [11] remains difficult, since their method operates directly on PCG recordings from a different dataset, while the present work estimates cardiac events from contactless radar-derived signals. Nevertheless, the results demonstrate significant improvements even with event-based evaluation, especially in the S2 $F_{1}$ score.

### 4.7. Computational Performance

To assess the practical feasibility of the proposed 1D U-Net architecture, presented in Section 3 and Section 4.4, for real-time operation, inference on the 4.0 s analysis window at a sampling frequency of 500 Hz has been performed.

In practice, we have evaluated the real-time factor (RTF), defined as the ratio between inference time and signal time duration, and the 95th and 99th percentile inference latency (i.e., $p_{95\mathrm{th}}$ and $p_{99\mathrm{th}}$), respectively, to identify the worst-case scenarios. To obtain steady-state metrics, a 15 segment warm-up sequence has been performed, before a 100-step inference run. This process has been repeated 100 times, with the aim of determining a mean ± standard deviation measurement for each figure of merit.

Our results, referring to both GPU (NVIDIA 4080) and CPU (AMD 7800X3D) inference, and shown in Table 10, suggest good real-time performance with a 1003× and 465× mean processing time gain (1/RTF) over real-time duration of the samples. The reported values of $p_{95\mathrm{th}}$ and $p_{99\mathrm{th}}$ standard deviation values also suggest consistent latency for both GPU and CPU inference.

From a deployment perspective, the proposed model is also relatively compact, since it contains 2,016,768 trainable parameters and requires only 7.69 MiB of memory, when float32 weights are considered, for model parameters. The low memory footprint and low RTF values, together with low $p_{95}\mathrm{th}$ and $p_{99}\mathrm{th}$ values, for the evaluated hardware, suggest a real-time embedded deployment for the developed architecture to aid in performing a patient’s contactless auscultation.

## 5. Conclusions

In this manuscript, the use of a self-attention 1D U-Net for the joint reconstruction of PCG waveforms and cardiac phase segmentation has been proposed. Our approach leverages a filtered radar displacement signal to separately capture HS components, enabling the reconstruction of a coherent PCG signal from a Doppler radar sensor.

The proposed NN output achieves substantial improvements in both spectral and temporal characteristics compared with the input radar signal, as evidenced by a reduction of 13.4885 dB in LSD. In addition, segmentation performance is significantly enhanced with respect to the PCG ground truth, yielding increases equal to 9.76% and 12.03% on average in Micro-$F_{1}$ and Macro-$F_{1}$ scores, respectively, and 0.0980 in envelope correlation, compared with baseline radar-based metrics. The ablation analysis demonstrates that the self-attention bottleneck substantially improves performance in both waveform reconstruction and cardiac state estimation.

These results highlight the feasibility of radar-based contactless auscultation and continuous cardiac monitoring, particularly in scenarios where direct physical contact is impractical or undesirable. Our assessment of specific computational metrics shows that the developed model is sufficiently lightweight to be deployable in low-power embedded systems for real-time applications, obtaining an over 1000× real-time speed gain with dedicated hardware GPU acceleration. Furthermore, the proposed approach benefits from inherent immunity to background acoustic noise, making it well suited for operation in busy environments.

Despite these improvements, reduced accuracy is observed in the detection of the S2 onset at the end of the systolic phase. This limitation is likely to originate from the lower relative amplitude of the second heart sound and the presence of respiratory artifacts. Degradation in evaluated metrics can also be observed during subject speech or movement. Future work will focus on enhancing the neural network robustness to RBM and respiratory interference, with the goal of improving both perceptual PCG reconstruction and segmentation accuracy.

Even if the presented results are promising, because of the limited dataset size and number of involved subjects, validation on a wider population, as well as across a wider range of conditions and environments, is required to ensure reliable operation in real-world settings. Addressing these aspects will be essential for compliance with medical device regulatory frameworks.

## Figures and Tables

**Table 1:** Brief description of the general self-attention 1D multitask U-Net architecture.

Stage	Configuration	Output Size
Input	3 input channels; 4.0 s window at 500 Hz	3×2000
Encoder	4 residual 1D encoding blocks; channels 24→48→96→192; stride-2 downsampling at each level	192×125
Bottleneck	1×1 projection to 192 channels; 1 transformer encoder layer with 8 heads; projection back to 192 channels	192×125
Decoder	4 decoding blocks with linear upsampling and skip connections; channels 192→96→48→24	24×2000
Refinement	1 residual 1D convolution block at 24 channels	24×2000
Output	3 task-specific heads: waveform, segmentation, and envelope	Waveform: 1×2000; Segmentation: 4×2000; Envelope: 1×2000

**Table 2:** Segmentation-state statistics from the dataset for radar and PCG. Values are reported as mean ± standard deviation.

Sensor	S1 Duration [s]	S1 Time [%]	Systole Duration [s]	Systole Time [%]	S2 Duration [s]	S2 Time [%]	Diastole Duration [s]	Diastole Time [%]
Radar	0.1046±0.0512	12.4296±7.7875	0.1578±0.0406	19.9162±5.0015	0.1221±0.0359	15.5773±5.2701	0.4201±0.1168	52.0770±8.9249
PCG	0.1226±0.0006	15.6595±2.5428	0.1937±0.0306	24.2872±3.0279	0.0920±0.0001	11.6816±1.9041	0.3899±0.0990	48.3718±6.1898

**Table 3:** Sensitivity-analysis metrics results. Values are reported as mean ± standard deviation; the best mean value for each metric is highlighted in bold.

Loss Weights α/β/γ	LSD [dB]	$r_{\mathrm{env}}$	Micro-$F_{1}$ [%]	Macro-$F_{1}$ [%]	S1 $F_{1}$ [%]	Systole $F_{1}$ [%]	S2 $F_{1}$ [%]	Diastole $F_{1}$ [%]
Radar input	21.7630±1.4444	0.5098±0.0870	77.04±4.76	70.08±6.01	60.86±8.62	75.04±5.42	56.36±7.70	88.07±2.30
0.80/0.10/0.10	9.2632±0.3864	0.5754±0.0908	87.13±3.18	83.08±3.89	84.18±3.12	86.93±3.69	68.90±6.90	92.30±2.22
0.70/0.20/0.10	9.3790±0.2254	0.5798±0.0936	88.02±3.05	84.11±3.86	85.15±3.30	87.83±3.47	70.46±7.05	93.02±1.91
0.60/0.30/0.10	9.3027±0.3297	0.5808±0.0922	88.00±3.30	84.11±4.17	84.94±4.36	87.70±3.70	70.67±7.15	93.13±1.93
0.50/0.40/0.10	9.5668±0.4205	0.5856±0.0933	87.83±3.51	83.87±4.42	84.37±4.82	87.40±3.89	70.70±7.22	93.02±2.19
0.45/0.45/0.10	9.5742±0.4521	0.5834±0.0963	87.77±3.38	83.73±4.33	84.55±4.29	87.30±3.85	70.03±7.51	93.06±2.01
0.40/0.50/0.10	9.6349±0.4392	0.5819±0.0957	88.03±3.34	84.08±4.23	84.79±4.25	87.70±3.74	70.68±7.21	93.15±2.07
0.33/0.33/0.33	9.7332±0.5740	0.5858±0.0926	87.25±4.06	83.20±4.97	83.47±5.45	86.64±4.72	70.11±7.62	92.56±2.69
0.30/0.60/0.10	9.6135±0.3391	0.5872±0.0955	87.64±3.09	83.59±3.95	83.80±4.30	87.19±3.47	70.31±6.52	93.08±1.72
0.20/0.70/0.10	9.7502±0.3893	0.5891±0.0969	87.74±3.25	83.73±4.12	83.91±4.53	87.28±3.73	70.59±6.71	93.15±1.77
0.10/0.80/0.10	9.8780±0.4213	0.5926±0.0983	87.94±3.31	83.90±4.32	84.58±4.54	87.81±3.63	70.10±7.66	93.11±1.86
0.10/0.10/0.80	9.6866±0.4931	0.5863±0.0931	87.75±3.26	83.66±4.20	84.16±4.06	87.53±3.58	69.95±7.46	93.01±1.87

**Table 5:** Training hyperparameters adopted for the proposed neural network model.

Hyperparameter	Value
Optimizer	AdamW
Learning rate	$5\cdot 10^{-5}$
Weight decay	$3\cdot 10^{-5}$
Number of epochs	50
Batch size	32
Input window duration	4 s
Sampling frequency	500 Hz
Input sequence length	2000 samples
Training/validation/test split	9/1/1 subjects
Loss weights α/β/γ	0.70/0.20/0.10

**Table 6:** Results grouped by test subject. Values are reported as mean ± standard deviation.

Subject	LSD [dB]	$r_{\mathrm{env}}$	Micro-$F_{1}$ [%]	Macro-$F_{1}$ [%]	S1 $F_{1}$ [%]	Systole $F_{1}$ [%]	S2 $F_{1}$ [%]	Diastole $F_{1}$ [%]
Subject 1	9.1261±0.2422	0.5901±0.0128	83.48±0.48	79.59±0.51	80.61±0.54	81.52±0.90	66.72±0.65	89.53±0.38
Subject 2	8.8436±0.1066	0.7099±0.0048	92.13±0.36	89.41±0.46	89.44±0.83	92.42±0.26	80.35±0.65	95.43±0.26
Subject 3	10.8434±0.4616	0.5597±0.0100	83.04±1.15	81.79±1.15	84.03±1.63	82.90±1.41	73.60±1.91	86.61±1.19
Subject 4	9.9308±0.6096	0.4836±0.0102	76.37±0.97	72.65±0.90	74.55±1.52	74.52±0.94	58.00±0.75	83.53±0.97
Subject 5	10.4459±0.1671	0.4136±0.0084	78.23±1.19	71.19±1.15	70.73±1.77	75.08±1.23	52.46±1.02	86.51±1.05
Subject 6	9.4958±0.0806	0.5745±0.0091	82.43±0.49	78.90±0.60	80.10±0.80	80.74±0.47	66.34±1.00	88.44±0.30
Subject 7	9.8724±0.1966	0.6125±0.0074	86.96±0.58	82.35±0.61	81.79±0.80	85.56±0.71	69.50±0.89	92.54±0.53
Subject 8	9.6019±0.2009	0.5076±0.0076	85.49±0.92	80.87±0.99	81.88±2.15	84.72±0.95	65.03±0.44	91.85±0.72
Subject 9	9.7265±0.1550	0.7079±0.0107	87.06±1.33	83.37±1.66	80.11±1.95	86.78±1.30	74.85±2.61	91.75±0.89
Subject 10	8.9445±0.1127	0.6900±0.0091	86.29±0.56	84.77±0.56	88.41±0.69	85.90±0.79	74.84±0.54	89.93±0.55
Subject 11	9.5835±0.3948	0.6315±0.0114	84.41±0.89	79.83±1.06	76.21±1.50	82.82±0.76	69.81±2.03	90.49±0.68
Aggregate	9.6740±0.6388	0.5892±0.0921	84.17±4.23	80.43±5.01	80.71±5.48	83.00±5.01	68.32±7.77	89.69±3.26
Radar input	23.1625±1.3302	0.4912±0.0755	74.41±3.93	68.40±4.67	63.57±6.76	70.93±5.94	54.43±7.31	84.66±2.95
Improvement	13.4885±1.3707	0.0980±0.0443	9.76±3.48	12.03±3.91	17.14±7.54	12.07±3.99	13.89±5.49	5.03±2.57

**Table 7:** Results grouped by acquisition scenario. Values are reported as mean ± standard deviation.

Scenario	LSD [dB]	$r_{\mathrm{env}}$	Micro-$F_{1}$ [%]	Macro-$F_{1}$ [%]	S1 $F_{1}$ [%]	Systole $F_{1}$ [%]	S2 $F_{1}$ [%]	Diastole $F_{1}$ [%]
Resting	9.4844±0.5188	0.6075±0.1088	85.40±5.84	81.85±6.39	80.88±7.92	84.66±6.43	71.33±8.34	90.54±4.94
After sport	9.6894±0.7403	0.6202±0.0898	87.74±5.76	84.25±7.08	84.64±6.49	86.35±7.00	73.47±12.23	92.56±3.97
Apnea	9.2835±0.9855	0.6095±0.1991	86.45±9.32	83.10±9.80	85.28±13.89	85.79±9.99	69.95±12.73	91.40±7.71
Apnea after sport	8.7906±0.3395	0.6917±0.0994	88.85±2.95	85.56±2.45	85.24±2.64	87.71±3.81	75.76±1.42	93.51±2.25
Angle variation	9.4796±0.8971	0.6140±0.1428	85.00±8.99	81.06±11.56	82.37±12.05	84.05±10.41	67.82±19.14	90.01±6.44
Distance variation	10.2696±0.6968	0.4182±0.1652	76.87±9.98	71.06±12.14	72.07±14.20	74.56±11.76	53.25±16.93	84.38±7.47
Speaking	15.6378±3.6841	0.6195±0.0938	80.33±9.19	76.71±11.84	79.08±13.21	78.37±11.34	63.25±17.57	86.15±6.58
Movement	9.2255±0.9109	0.5388±0.1678	77.60±16.45	72.99±18.46	69.25±23.64	75.47±18.18	63.02±19.29	84.24±13.00
Standing	9.6584±0.7230	0.5918±0.1121	80.99±7.53	78.06±7.61	80.75±8.06	79.64±8.45	65.67±9.99	86.16±7.73
Lying	9.0076±0.1817	0.6654±0.0164	87.57±0.92	83.24±1.40	79.85±2.43	88.07±0.87	71.47±2.67	93.59±0.33

**Table 8:** Per-class precision, recall, and $F_{1}$ values for the runs. Values are reported as mean ± standard deviation.

State	Precision [%]	Recall [%]	$F_{1}$ [%]
S1	78.61±6.14	83.03±5.36	80.71±5.48
Systole	83.96±6.04	82.20±5.10	83.00±5.01
S2	66.82±7.25	69.92±8.49	68.32±7.77
Diastole	90.43±3.60	89.00±3.44	89.69±3.26

**Table 9:** Event-based S1 and S2 detection results using ECG-derived reference events and a ±100 ms tolerance window. Values are reported as mean ± standard deviation.

Method	Input Modality	S1 $F_{1}$ [%]	S2 $F_{1}$ [%]	S1+S2 $F_{1}$ [%]
Springer et al. [11]	PCG	96.95±0.90	94.29±1.08	95.63±0.85
Radar input baseline	Radar-derived HS signal	87.12±6.70	79.35±10.77	83.23±7.66
Proposed self-attention 1D U-Net	Radar-derived HS signal	86.73±5.57	88.61±5.64	87.65±5.55

**Table 10:** Comparison between NVIDIA 4080 (GPU) and AMD 7800X3D (CPU) in terms of RTF, $p_{95\mathrm{th}}$ and $p_{99\mathrm{th}}$.

Hardware	RTF	$p_{95}\mathrm{th}$ [ms]	$p_{99}\mathrm{th}$ [ms]
NVIDIA 4080	$\left(0.997\pm 0.022\right)\cdot 10^{-3}$	5.225 ± 0.522	6.362 ± 0.960
AMD 7800X3D	$\left(2.149\pm 0.028\right)\cdot 10^{-3}$	12.112 ± 0.806	17.209 ± 1.915

## Data Availability

The datasets analyzed during the current study are publicly available on Figshare at https://doi.org/10.6084/m9.figshare.9691544.v1, and are associated with the publication cited in the reference list (Ref. [24]).

## Abbreviations

The following abbreviations are used in this manuscript:

BR	Breath Rate
CAT	Computerized Axial Tomography
CE	Cross-Entropy
CS	Contactless Stethoscope
CW	Continuous Wave
DACM	Differentiate and Cross-Multiply
ECG	Electrocardiogram
FFT	Fast Fourier Transform
GELU	Gaussian Error Linear Unit
GAN	Generative Adversarial Network
HR	Heart Rate
HS	Heart Sounds
HSMM	Hidden Semi-Markov Model
I/Q	In-phase and Quadrature
LSD	Log-Spectral Distance
MAE	Mean Absolute Error
MRI	Magnetic Resonance Imaging
NN	Neural Network
PCG	Phonocardiogram
PPG	Photoplethysmogram
RBM	Random Body Movement
RTF	Real-Time Factor
SCG	Seismocardiogram
SISO	Single-Input Single-Output
STFT	Short-Time Fourier Transform
VSM	Vital Signs Monitoring
